# Mitochondrial Genome Sequencing Reveals *orf463a* May Induce Male Sterility in NWB Cytoplasm of Radish

**DOI:** 10.3390/genes11010074

**Published:** 2020-01-09

**Authors:** Yanping Wang, Qingbiao Wang, Wei Hao, Jianxia Li, Meixia Qi, Li Zhang

**Affiliations:** National Engineering Research Center for Vegetables, Beijing Academy of Agriculture and Forestry Sciences, Key Laboratory of Biology and Genetic Improvement of Horticultural Crops (North China), Beijing Key Laboratory of Vegetable Germplasm Improvement, Haidian District, Beijing 100097, China; wangyanping@nercv.org (Y.W.); wangqingbiao@nercv.org (Q.W.); 15076205291@163.com (W.H.); Radish312@163.com (J.L.); qimeixia0702@163.com (M.Q.)

**Keywords:** cytoplasmic male sterility, DCGMS, NWB CMS, *orf463a*, radish

## Abstract

Radish (*Raphanus sativus* L.) is an important root vegetable worldwide. The development of F1 hybrids, which are extensively used for commercial radish production, relies on cytoplasmic male sterility (CMS). To identify candidate genes responsible for CMS in NWB cytoplasm, we sequenced the normal and NWB CMS radish mitochondrial genomes via next-generation sequencing. A comparative analysis revealed 18 syntenic blocks and 11 unique regions in the NWB CMS mitogenome. A detailed examination indicated that *orf463a* was the most likely causal factor for male sterility in NWB cytoplasm. Interestingly, *orf463a* was identical to *orf463*, which is responsible for CMS in Dongbu cytoplasmic and genic male sterility (DCGMS) radish. Moreover, only structural variations were detected between the NWB CMS and DCGMS mitochondrial genomes, with no nucleotide polymorphisms (SNPs) or meaningful indels. Further analyses revealed these two mitochondrial genomes are coexisting isomeric forms belonging to the same mitotype. *orf463a* was more highly expressed in flower buds than in vegetative organs and its expression was differentially regulated in the presence of restorer of fertility (*Rf*) genes. *orf463a* was confirmed to originate from *Raphanus raphanistrum*. In this study, we identified a candidate gene responsible for the CMS in NWB cytoplasm and clarified the relationship between NWB CMS and DCGMS.

## 1. Introduction

Radish (*Raphanus sativus* L.), which is a Brassicaceae crop, is mainly used as a root vegetable and is widely cultivated in East Asia, especially in China, Japan, and South Korea [1]. As a cross-pollinated plant, radish exhibits high hybrid vigor, which is beneficial for the commercial production of hybrid seeds. Self-incompatibility and cytoplasmic male sterility (CMS) are two major traits associated with radish hybrid seed production. However, because of their vulnerability to environmental factors, self-incompatible lines are unstable, which results in the production of impure hybrid seeds. Fortunately, male sterility may be useful for developing an efficient and cost-effective method for applying heterosis to hybrid production.

Cytoplasmic male sterility is a maternally inherited trait in which plants develop normally, but fail to produce functional pollen, and is often associated with the rearrangement of genes in the mitochondrial genome [2,3]. Plant mitochondrial genomes, which differ considerably from animal mitochondrial genomes, are large (200–2400 kb), complex, and comprise multipartite structures because of the recombination and nonhomologous end-joining (NHEJ) activities which result in formation of novel open reading frame (*orf*) genes [2,4]. These genes often share chimeric structures with known mitochondrial genes and encode proteins with transmembrane domains. The accumulation of such novel chimeric ORFs usually leads to CMS, resulting in the abnormal development of floral organs or pollen grains [2,5]. The fertility restorer (*Rf*) genes are nuclear genes that have been transported into the mitochondria, wherein they inhibit the expression of novel *orf* genes at different levels to compensate for mitochondrial dysfunction and restore fertility [6,7,8,9,10].

Cytoplasmic male sterility has been observed in many plant species, including some economically important crops such as rice, maize, and sorghum [11,12]. Ogura CMS, which was identified by a Japanese researcher [13], was the first confirmed example of CMS in radish. The mitochondrial gene responsible for Ogura male sterility is *orf138*, which is specific to the mitochondrial genomes of various radishes with an Ogura-type cytoplasm [14]. Although Ogura CMS has been widely used for breeding Brassicaceae crops [15,16], the high genetic uniformity in the cytoplasm may lead to disease susceptibility, which has necessitated the identification and characterization of new types of CMS [17]. Nahm et al. revealed another CMS in radish, NWB CMS, which was detected in a Korean radish collection and differs from Ogura CMS [18]. The NWB CMS radish plants produce more yellowish anthers than the Ogura CMS lines as well as some non-viable pollen grains. Moreover, an analysis of NWB CMS radish plants by PCR with a primer pair specific for the Ogura *orf138* gene resulted in no amplification product. A primer pair targeting the 3′ region of the *atp6* gene and the 5′ region of the *nad3* gene was developed as a molecular marker specific for the NWB CMS line [18]. More recently, a new radish CMS resulting in a phenotype similar to that of NWB CMS was observed in an accession collected in Uzbekistan and was subsequently designated as Dongbu cytoplasmic and genic male sterility (DCGMS) [19]. An amplicon was detected during a PCR analysis of the DCGMS line with a primer pair designed for NWB CMS radish [19], implying that NWB CMS and DCGMS may be the same CMS type. However, the exact relationship between NWB CMS and DCGMS has not been determined. Advances in high-throughput next-generation sequencing have provided researchers with an experimental technique that enables the rapid and efficient identification of candidate mitochondrial genes responsible for male sterility. The DCGMS mitochondrial genome has been fully sequenced and a novel chimeric gene, *orf463*, was identified as the most likely gene responsible for DCGMS [20]. However, the factor mediating the CMS in the NWB CMS line remains unknown. Thus, the potential relationship between NWB CMS and DCGMS must be explored further.

In this study, we applied a next-generation sequencing approach to identify the genes associated with NWB CMS. We determined and compared the complete nucleotide sequences and organization of the mitochondrial genomes of normal and NWB CMS radish lines. We also screened for candidate *orf* genes inducing male sterility in NWB CMS radish. Furthermore, the relationship between NWB CMS and DCGMS was clarified based on analyses of phylogeny, synteny, single nucleotide polymorphisms (SNPs), and insertion–deletion (indel) mutations.

## 2. Materials and Methods

### 2.1. Plant Materials

We obtained YB, which is an open-pollination variety with white flesh and a round root, from southern China. The CR301 hybrid purchased from Beijing Shinong Seed Co. (i.e., subsidiary of Noo Woo Bio Co., Korea) was confirmed as male sterile with the NWB cytoplasm based on an analysis with the NWB-specific primers designed by Nahm et al. in 2005 [18]. To obtain the male-sterile line YB-A with the NWB cytoplasm and its maintainer line YB-B, YB was crossed with CR301 and backcrossed eight times, with YB as the paternal line in all crosses. The mitochondrial DNA of YB-A (i.e., NWB CMS) and YB-B (i.e., normal) was sequenced.

To assess the specificity of seven unique *orf* genes associated with NWB cytoplasm, the following 12 radish varieties were analyzed: YB-A, YB-B, Duanye 13 (South China), Qingbaoxia (Northwest China), YR Tengu (Japan), Xixing No. 5 (East China), Weiqing (East China), JZ Wujinhong (Northeast China), Manshenhong (West China), Banye Chunlihong (East China), Mantanghong (North China), and Gang Shui (Northeast China). With the exception of YB-A, all varieties contained normal cytoplasm and lacked *Rf* genes (Table S1). To investigate the distribution of *orf463*, *orf463*-specific primers [20,21] were used to amplify the DNA from 93 radish accessions representing natural populations [22].

To determine the *orf463a* expression profile in the presence of *Rf* genes, YB-A was crossed with three inbred lines (Hongbaoshi, Fure, and Rudi) containing *Rf* genes. Three F_2_ populations were obtained via the self-pollination of three fertile F_1_ plants (Table S1). The expression of *orf463a* was analyzed in three F_2_ populations (96 SA18-14 plants, 98 SA18-15 plants, and 98 SA18-16 plants), three fertile plants (three replicates for each plant) in each F_2_ population were randomly selected. Details regarding the primers used for PCR amplifications are presented in Table S2.

### 2.2. Mitochondrial DNA Sequencing and Genome Assembly

Mitochondrial DNA was isolated from approximately 5 g etiolated leaves as described by Chen et al. [23], after which 1 μg purified DNA was fragmented to construct short-insert libraries (insert size 430 bp) with the TruSeq™ Nano DNA Sample Prep Kit (Illumina, San Diego, CA, USA). The libraries were then sequenced with the Illumina HiSeq 4000 system [24]. The high molecular weight DNA was purified and used to prepare PacBio libraries. The BluePippin Size-Selection system was used to prepare size-selected libraries, which were then sequenced with the Sequel Sequencer. Illumina raw reads were first filtered to remove reads with adapters, reads with a low quality score (Q < 20) or with ≥10% uncalled bases, and duplicated reads. The mitochondrial genome was reconstructed with a combination of the PacBio Sequel data and the Illumina HiSeq data. Specifically, the genome framework was assembled based on the Illumina and PacBio data with SPAdes (version 3.10.1) [25]. There were 8 contigs (84,279 bp, 44,451 bp, 42,651 bp, 41,440 bp, 33,600 bp, 18,013 bp, 10,927 bp and 726 bp, respectively) generated by de novo assembly, the order and direction of contigs were determined by the overlap and repeat sequences. Next, the assembled mitochondrial genome was checked to complete the circle. Clean reads were then mapped to the assembled mitochondrial genome to verify the accuracy of the sequence. Insertions and deletions were also detected.

### 2.3. Genome Annotation

Mitochondrial genes were annotated based on sequence homology. De novo predicted gene sets were integrated with EVidenceModeler (version 1.1.1) [26]. The tRNA and rRNA genes were predicted with tRNAscan-SE and rRNAmmer 1.2 [27,28]. The circular mitochondrial genome map was drawn with OrganellarGenomeDRAW (version 1.2) [29].

### 2.4. Construction of a Phylogenetic Tree, Prediction of Transmembrane Domains, and Sequence Comparisons

Conserved 40-kb sequence blocks from eight radish mitotypes [17] were extracted from the NCBI database, after which a BLAST search was used to detect matches between these sequences and our normal and NWB CMS mitotype sequences. The identified conserved sequences were aligned with ClustalW. A phylogenetic tree was constructed with MEGA X [30]. Specifically, the neighbor-joining method was used [31]. The evolutionary distances were calculated with the Maximum Composite Likelihood method [32]. Transmembrane domain-encoding sequences in the *orf* gene were predicted with the TMHMM server (version 2.0) [33]. The synteny among the whole genomes of NWB CMS, DCGMS, and black radish (AP012990) lines was analyzed with MUMmer (version 3.23) and LASTZ (version 1.03.54). Additionally, SNPs were detected with MUMmer and BLAT (version 35), whereas indels were identified with LASTZ (version 1.03.54), BWA, and SAMtools.

### 2.5. RNA Extraction, RT-PCR, Real-Time PCR, and Amplicon Sequencing

We collected leaves, stems, and floral buds at different pollen developmental stages. Floral buds <2 mm and 5 mm corresponded to pollen developmental stages before and after the microspore stage, respectively [34]. Total RNA was extracted from the collected samples with the Quick RNA isolation Kit (Huayueyang Biotechnology, Beijing, China). Total RNA quantity and quality were checked with the NanoDrop 1000 spectrophotometer (Thermo Fisher Scientific Inc., Waltham, MA, USA) and by 1% non-denaturing agarose gel electrophoresis, respectively. The total RNA (2 μg) was used as the template to synthesize cDNA with the FastKing RT Kit (with gDNase) (Tiangen Biotech, Beijing, China). The obtained cDNA was diluted in 100 μL and then used as the template for qRT-PCR assays, which were completed with the LightCycler 480 Real-Time PCR system (Roche, Basel, Switzerland) with gene-specific primers (Supplementary Table S2). Each reaction consisted of 10 μL SYBR Green I Master Mix, 2 μL cDNA, 1 μL primer mix (10 μM each primer), and 7 μL ddH_2_O for a final volume of 20 μL. The qRT-PCR program was as follows: 95 °C for 5 min; 40 cycles of 95 °C for 20 s, 60 °C for 20 s, and 72 °C for 20 s. The PCR amplification of the expected *orf463a* sequence was confirmed by agarose gel electrophoresis and sequencing. RNA polymerase-II transcription factor was used to standardize each reaction run with respect to RNA integrity, sample loading, and inter-PCR variations [35]. Relative gene expression levels were calculated according to the 2^−ΔΔCt^ method. The PCR program was as follows: 94 °C for 5 min; 35 cycles of 94 °C for 30 s, 57 °C for 30 s, and 72 °C for 1 min; 72 °C for 10 min. The amplified products were detected by electrophoresis on a 1% agarose gel (containing GoldView nucleic acid dye) and analyzed with the UVI gel imaging system. The correct product was sequenced by TsingKeBioTech, Beijing, China.

## 3. Results

### 3.1. Mitochondrial Genome Organization of NWB CMS and Normal Radish

**Figure 1:**
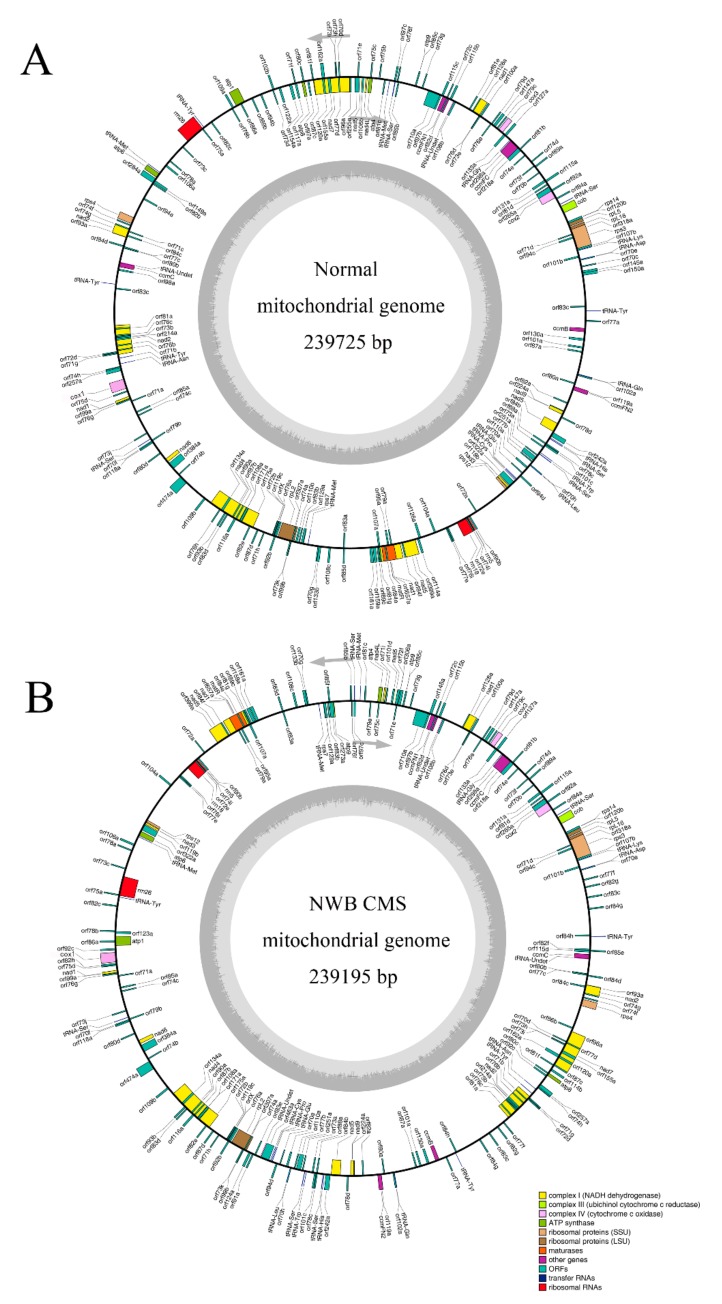
Organization of the normal (**A**) and NWB CMS (**B**) mitochondrial genomes. Genes on the forward and reverse strands are presented on the outside and inside of the circle, respectively. The two gray layers represent the GC content in the mitochondrial genome.

The NWB CMS and normal radish mitochondrial genomes were sequenced with the Illumina HiSeq and PacBio sequencing systems and assembled into single circular sequences comprising 239,195 bp (NCBI accession: MN056360) and 239,725 bp (NCBI accession: MN056359), respectively. The GC content of the NWB CMS and normal radish mitochondrial genomes was 45.13% and 45.26%, respectively, which was similar to that of other mitochondrial genomes from Cruciferae species (Figure 1, Table S3). Additionally, the non-coding RNAs (i.e., tRNA and 5S, 18S, and 26S rRNAs) in the mitogenomes were predicted with tRNA scan-SE (version 1.3.1) and by screening for homology. The non-coding RNA types, average length, and total length were similar between the NWB CMS and normal mitochondrial genomes, but an additional tRNA for MET was detected in the NWB CMS mitogenome (Tables S4 and S5).

The NWB CMS mitochondrial genome included the 33 protein-coding genes present in the normal mitochondrial genome, but also contained an additional gene, *ATP9-D2* (Figure 1, Table S6). A comparison of amino acid sequences indicated that ATP9 and ATP9-D2 differ only at amino acid position 22, with an isoleucine and valine in ATP9-D2 and ATP9, respectively (Figure S1). This difference was consistent with the difference encoded in the two *atp9* genes in the DCGMS mitotype [20]. In contrast, the Ogura mitotype comprises two identical *atp9* genes [36]. Among the 33 conserved protein-coding genes, 18 encode components of the electron transport chain and ATP synthase, including nine subunits of complex I (*nad1*, *nad2*, *nad3*, *nad4*, *nad4L*, *nad5*, *nad6*, *nad7*, and *nad9*), one subunit of complex III (*cob*), three subunits of complex IV (*cox1*, *cox2*, and *cox3*), and five subunits of complex V (*atp1*, *atp4*, *atp6*, *atp8*, and *atp9*). Additionally, five genes are involved in the biogenesis of cytochrome C (*ccmB*, *ccmC*, *ccmFN1*, *ccmFN2*, and *ccmFC*), eight genes encode ribosomal proteins (*rpl2*, *rpl5*, *rpl16*, *rps3*, *rps4*, *rps7*, *rps12*, and *rps14*), and two genes encode maturase (*matR*) and orfX.

### 3.2. Identification of Sequence Variations in the Genes Encoding Known Proteins between the NWB CMS and Normal Mitochondrial Genomes

The NWB CMS and normal mitochondrial gene sequences were compared to detect mutations possibly related to CMS (Table 1). Sequence analyses detected SNPs, but no indels, in seven known protein-coding genes between the normal and NWB CMS mitochondrial genomes. The two SNPs in *coxI* and the single SNP in *rps12* were synonymous mutations. However, the single SNPs in *rps3*, *rps4*, and *nad7* resulted in amino acid differences, which was consistent with what was reported for the DCGMS mitochondrial genome [20]. Two and six SNPs were identified in *matR* and *rpl2*, respectively, including synonymous and nonsynonymous mutations.

### 3.3. Screening for Specific orf Genes Responsible for the Male Sterility in NWB CMS

**Figure 2:**
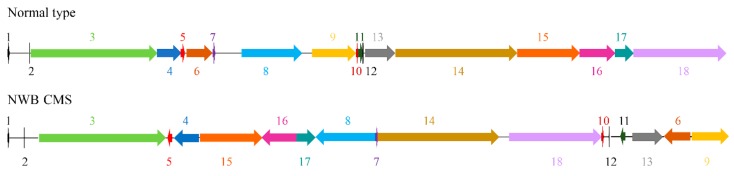
Comparison of the location and orientation of 18 syntenic regions between the NWB CMS and normal mitochondrial genomes.

A total of 18 syntenic blocks and 11 unique regions were identified in the NWB CMS mitogenome via a comparison of the complete NWB CMS and normal mitochondrial genome sequences (Figure 2). The position and orientation of these syntenic blocks were considerably rearranged in the NWB CMS mitotype, usually because of the homologous recombination mediated by short repeats (<1 kb). Further analyses revealed nine short repeats in the NWB CMS mitogenome, of which R1, R5, R6, R7, and R9 were located around the junctions between syntenic sequence blocks (Table 2). Additionally, both mitotypes had large repeats (>1 kb), with two copies of 9731 bp and 5909 bp sequences in the NWB CMS and normal mitochondrial genomes, respectively.

**Figure 3:**
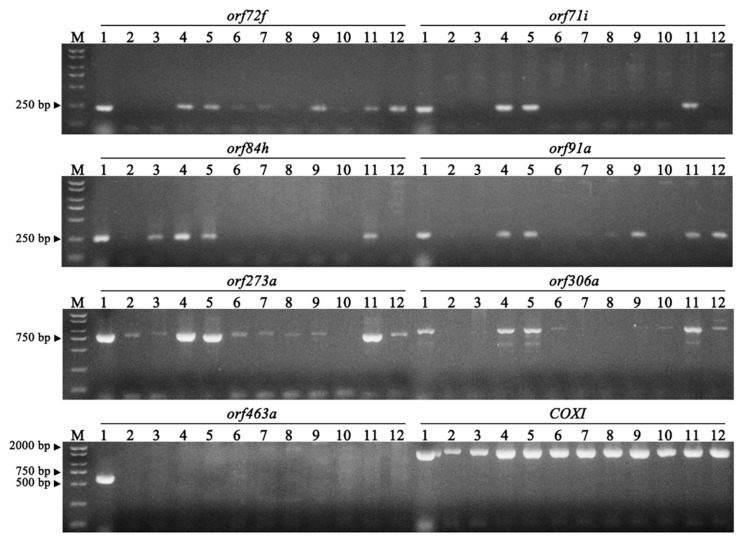
Amplification of seven candidate *orf* genes in 12 representative varieties. The *CoxI* gene was used as a positive control. Lanes 1–12 correspond to the following samples: YB-A, YB-B, Duanye 13 (South China), Qingbaoxia (Northwest China), YR Tengu (Japan), Xixing No. 5 (East China), Weiqing (East China), JZ Wujinhong (Northeast China), Manshenhong (West China), Banye Chunlihong (East China), Rudi (Netherlands), and Gang Shui (Northeast China).

Because the formation of new *orf* genes due to active rearrangements of the mitochondrial DNA is considered to be responsible for CMS, the *orf* genes in the NWB CMS and normal mitotypes encoding proteins longer than 70 amino acids were investigated. The *orf* genes were named according to the number of encoded amino acids. We identified 196 and 195 *orf* genes in the NWB CMS and normal mitogenomes, respectively. Moreover, the NWB CMS mitogenome had 16 unique *orf* genes, of which 12 were in the unique region and four were in the non-unique region. These genes were further examined to determine whether they were chimeric with known mitochondrial genes or encoded transmembrane structures (Table 3). Four of the unique *orf* genes (*orf84h*, *orf306a*, *orf273a*, and *orf463a*) encoded transmembrane structures, which is a very important feature of new *orf* genes inducing CMS. Moreover, five of the unique *orf* genes (*orf306a*, *orf72f*, *orf71i*, *orf273a*, and *orf91a*) were located upstream or downstream of known mitochondrial genes. Furthermore, *orf273a* and *orf463a* comprised partial sequences of known mitochondrial genes and unidentified sequences. Overall, there were seven candidate genes that encoded a transmembrane structure or were located upstream/downstream of known mitochondrial genes, of which only *orf463a* was unique to the NWB CMS mitogenome (Figure 3). Additionally, the *orf463a* sequence was identical to that of *orf463*, which is the most likely causal factor of CMS in DCGMS radish, with 128 bp corresponding to part of the *COX1* sequence and a 1261-bp sequence that did not match any known mitochondrial genes.

### 3.4. The NWB CMS and DCGMS Mitogenomes Are Coexisting Isomeric Forms of the Same Mitotype

**Figure 4:**
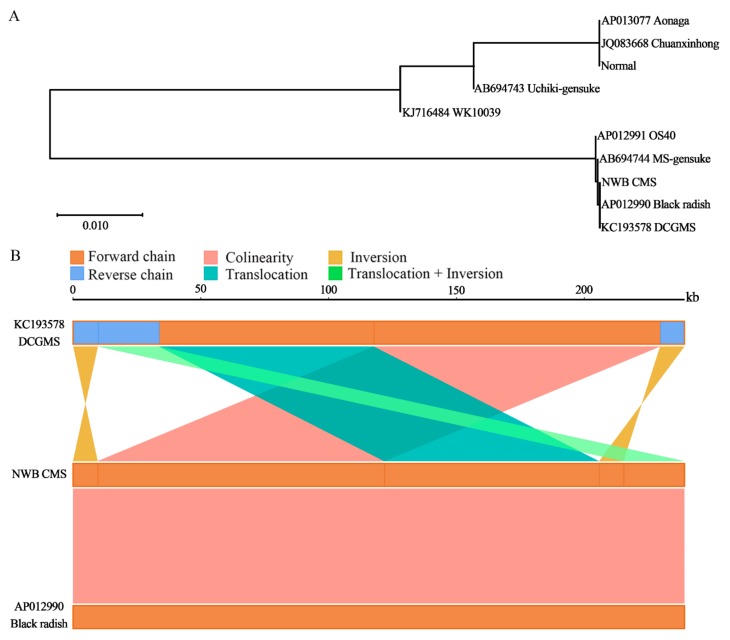
(**A**) Phylogenetic tree consisting of 10 radish mitotypes. The tree was constructed based on approximately 40-kb syntenic blocks. The optimal tree with a branch length sum of 0.129 is presented. The phylogenetic tree is drawn to scale, with branch lengths in the same units as those of the evolutionary distances used to construct the tree. (**B**) Analysis of syntenic sequences among the NWB CMS, DCGMS, and black radish (AP012990) lines. The three parallel bars represent the mitochondrial genomes. Dark-orange and blue regions in each bar represent the forward and reverse directions of the aligned genome, respectively. Lines between the two bars indicate the synteny types and locations as follows: magenta, blue-green, dark yellow, and light-green represent collinearity, translocation, inversion, and translocation + inversion, respectively.

To evaluate the relationship between the NWB CMS and DCGMS lines, we compared them regarding phylogenetic relationships, syntenic sequence similarities, and the presence of SNPs and indels. To assess the phylogenetic relationships, approximately 40 kb syntenic blocks from the eight sequenced mitochondrial genomes of different radish cultivars [17] were used to construct a phylogenetic tree. The NWB CMS and normal mitotypes were on different branches. Additionally, the NWB CMS line was very closely related to the DCGMS line and black radish (AP012990), whereas the normal mitotype was more closely related to Chuanxinhong and Aonaga (Figure 4A, Table S6).

**Figure 5:**
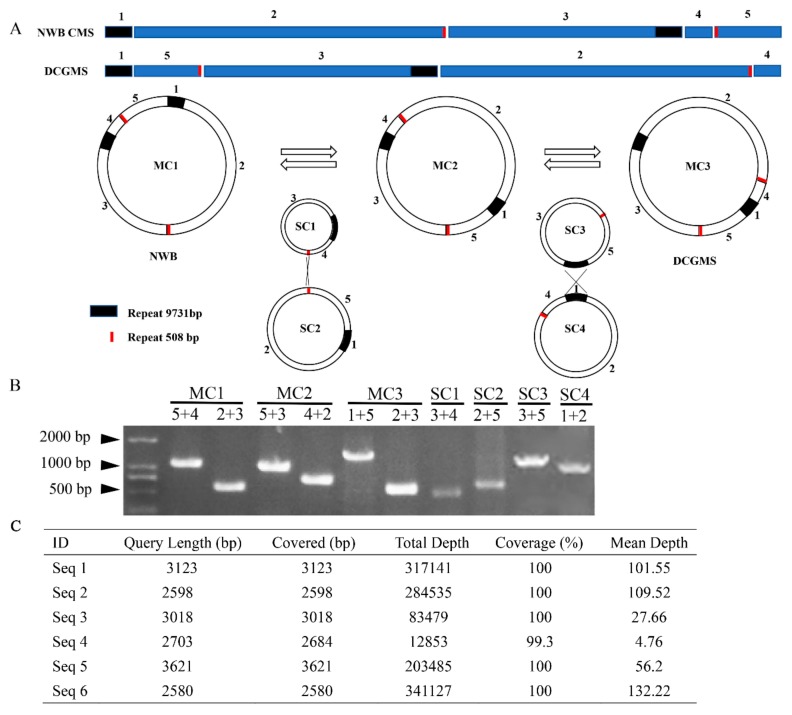
Multiple organizations of the NWB CMS mitochondrial genome. (**A**) Schematic diagram presenting the five syntenic blocks between the NWB CMS and DCGMS mitochondrial genomes, the three convertible master circles (MC), and the combinable subgenomic circles (SC). All circles are drawn to scale. Cross lines between SC1 and SC2 and between SC3 and SC4 indicate the recombination locations. The filled boxes represent repeat sequences. (**B**) PCR-amplified products with primers annealing to sequence blocks around the junctions. (**C**) The PacBio sequencing data for Seq 1 to Seq 6. Seq 1 to Seq 6 means respectively the sequence of MC1(5 + 4)/MC1(2 + 3)/MC2(5 + 3)/MC2(4 + 2)/MC3(1 + 5)/MC3(2 + 3) amplification plus its upstream and downstream 1000 bp. The sequence blocks in panel (**A**) targeted by the primers are indicated as follows: MC1 (5 + 4 and 2 + 3), MC2 (5 + 3 and 4 + 2), MC3 (1 + 5 and 2 + 3), SC1 (3 + 4), SC2 (2 + 5), SC3 (3 + 5), and SC4 (1 + 2).

To further clarify the relationships among the NWB CMS, DCGMS, and black radish (AP012990) lines, we analyzed the similarities in syntenic sequences, with the NWB CMS sequence used as a reference. A high degree of collinearity was observed between the NWB CMS and black radish (AP012990) sequences (Figure 4B), reaching 100%, whereas two inversions, one translocation, and one translocation + inversion were detected between the NWB CMS and DCGMS sequences. A previous study confirmed that the NWB CMS mitochondrial genome is very similar in size to the DCGMS mitochondrial genome (239,186 bp) [20]. A detailed analysis of sequence variations revealed a lack of SNPs among the three mitochondrial genomes. However, some indels were detected, with almost all of them located in poly-A, -T, or -G sequences, possibly because of a sequencing bias. Our results implied that the NWB CMS and DCGMS mitochondrial genomes were differentially organized, even though their sequences were nearly identical. Thus, there may be diverse isomeric master circles in the NWB CMS mitochondrial genome, similar to that in the Ogura CMS mitochondrial genome [20], which are likely due to recombination events mediated by repeat sequences. To verify this, we completed PCR amplifications with primers specific for sequence blocks flanking the recombination breakpoints. The resulting PCR data demonstrated that the NWB CMS mitochondrial genome may comprise three isomeric master circles (Figure 5A). Additionally, merging the subgenomic circles SC1 and SC2 in two possible orientations might produce the isomeric master circles MC1 (the form in the NWB CMS mitochondrial genome) and MC2 (Figure 5A), whereas merging SC3 and SC4 in two possible orientations might produce the isomeric master circles MC2 and MC3 (reportedly the form in the DCGMS mitochondrial genome). Furthermore, PCR amplifications with primers flanking the boundaries of SC1, SC2, SC3, and SC4 also proved the existence of these four putative subgenomic circles (Figure 5B). These genomic organizations were confirmed by sequencing the PCR products. Moreover, to avoid the limitation of PCR-based detection of recombination via large repeats, we present the ratio between recombinants using some specific long reads generated by PacBio sequencing (Figure 5C). Our result showed that there were indeed three isoforms of mtDNA molecules exist in the NWB mitochondrial genome. MC1 and MC3 seemed to be the dominant form. However, only MC1 could be assembled into a circle form which we presented in this study, and MC3 was assembled into a linear form in our de novo assembling.

### 3.5. Expression of orf463a in the Absence or Presence of Rf Genes

**Figure 6:**
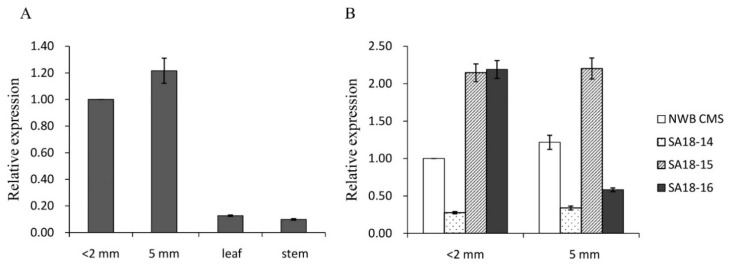
(**A**) Expression of *orf463a* in flower buds at different developmental stages (<2 mm: before the microspore stage; 5 mm: after the microspore stage) as well as in the leaf and stem. (**B**) Expression of *orf463a* in the NWB CMS line and its restorer lines (SA18-14, SA18-15, and SA18-16) at different pollen developmental stages. Error bars on each point indicate ± SE from three independent replicates.

An analysis of the radish organ-specific expression of *orf463a* indicated this gene was more highly expressed in flower buds than in the stem or leaf (Figure 6A). Moreover, the *orf463a* expression level increased after the microspore stage, which was consistent with the results of an earlier cytological examination that revealed abnormal pollen abortion after the microspore stage [19]. To evaluate the regulatory effects of *Rf* genes on *orf463a* expression, fertile plants were randomly selected from three F_2_ populations (SA18-14, -15, and -16) and examined. The *orf463a* expression level was low in SA18-14 both before and after the microspore stage, whereas it was relatively high in SA18-15 (Figure 6B). Regarding SA18-16, the *orf463a* expression level was relatively high before the microspore stage, but it decreased considerably after the microspore stage (Figure 6B). These inconsistent results may have been due to differences in the number and mechanisms of *Rf* genes among the examined fertile F_2_ plants.

### 3.6. Distribution of orf463a in Various Radish Species

A PCR assay with *orf463*-specific primers [20] was completed to determine the distribution of *orf463a* in 93 radish accessions from six cultivated radish subspecies and three wild species (Table 4). This gene was detected in nine accessions (9.68% of the tested materials). A comparison of the *orf463a* sequences in these nine accessions proved that one wild radish (*Raphanus raphanistrum*) originating from Romania, six black Spanish radish accessions (*R. sativus* var. *niger* Kerner), and one East Asian long radish accession (*R. sativus* var. *hortensis* Becker) from Noo Woo Bio Co. (Korea) had identical *orf463a* sequences. In contrast, the corresponding sequence in one wild radish (*Raphanus maritimus*) originating from Germany contained nine nucleotide substitutions. Although *orf463a* was detected in both *R. raphanistrum* from Romania and *R. maritimus* from Germany, the fact the sequence in *R. raphanistrum* was identical to the *orf463a* in the tested radish cultivars suggests *orf463a* may have originated in the *R. raphanistrum* wild radish species in Romania.

## 4. Discussion

The traditional approach to identifying CMS-associated *orf* genes mainly relied on Northern blot analyses, in which transcript profiles were compared between CMS and fertility-restorer lines. Candidate *orf* genes were identified based on their altered expression patterns in the presence of a restorer gene [37]. To identify the causal factor of NWB CMS, we followed the methods of Tanaka et al. (2012) and Chang et al. (2013) [36,38] to compare the NWB CMS and normal mitochondrial genome sequences. Similar to other CMS systems [20,36], the structure of the NWB CMS mitogenome differed substantially from that of the normal mitogenome (Figure 2), due to recombination and nonhomologous end-joining (NHEJ) activities which result in formation of new *orfs* possibly including genes responsible for the CMS in NWB CMS. An examination of 12 natural populations indicated that *orf463a* is specific to the mitochondrial genomes of radish lines with the NWB-type cytoplasm, making it the most likely candidate gene inducing the CMS in NWB CMS lines (Figure 3). Although some point mutations in known mitochondrial genes were detected in the NWB CMS mitogenome (Table 1), the amino acid changes may not be related to the induction of male sterility in the NWB CMS mitotype. Since mutations to these important mitochondrial genes may induce growth defects, but there were no significant morphological differences between the NWB CMS and normal lines other than male sterility [20].

Interestingly, the *orf463a* in the NWB CMS mitochondrial genome was identical to *orf463*, which is likely responsible for the CMS in DCGMS lines. An earlier investigation proved that DCGMS and NWB CMS lines are phenotypically similar, with both producing non-viable pollen grains [19]. This similarity compelled us to further evaluate the relationship between NWB CMS and DCGMS. A phylogenetic tree constructed based on the largest sequence block of 10 sequenced mitotypes revealed the extremely close relationship among NWB CMS, black radish (AP012990), and DCGMS lines (Figure 4A). Additionally, an analysis of syntenic sequences suggested that NWB CMS and black radish (AP012990) mitogenomic sequences are identical, whereas the NWB CMS and DCGMS mitogenomes contain some structural variations. A sequence comparison revealed a lack of SNPs among these three mitogenomes and the detected indels were all in poly-A, -T, or -G regions. Our data appear to indicate that the NWB CMS and black radish (AP012990) mitogenomes are identical. Yamagishi et al. recently reported that black radish (AP012990) and DCGMS lines carry coexisting mitogenomes and they hypothesized that the mechanism underlying the rearrangement of these two mitochondrial types is mediated by the 9730-bp and 512-bp repeats [21]. However, these possible recombinations were not verified by PCR. We designed primers specific for sequences around the block junctions and proved the existence of different forms of master circles as well as the possible recombinations of subgenomic circles of the NWB CMS mitogenome. One recombination type is exactly the same as that detected in the DCGMS mitogenome. Overall, these findings imply that the CMS of NWB CMS and DCGMS lines is the same and the associated mitogenomes are two coexisting isomeric forms of the same mitotype.

Regarding the origin of *orf463a*, we confirmed the presence of this gene in *R. raphanistrum* and *R. maritimus* (i.e., wild radish) (Table 4) which are possible ancestors of R. *satvius* [22,39]. A sequence analysis indicated that the *orf463a* sequence in *R. maritimus* contains nine nucleotide substitutions, seven of which are nonsynonymous. This is consistent with the recent results of a study of RS-5 in *R. raphanistrum* [21]. However, we revealed that the *orf463a* in *R. raphanistrum* is identical to the *orf463a* sequences in the mitogenomes of the NWB CMS line, six black Spanish radish accessions (*R. sativus* var. *niger* Kerner), and one East Asian long radish accession (*R. sativus* var. *hortensis* Becker). In our previous study regarding the genetic diversity and evolutionary relationships among *Raphanus* species, both M117 and M112 were classified as *R. raphanistrum*, whereas they belonged to different groups in the UPGMA dendrogram [22]. The *R. raphanistrum* RS-5 used by the Japanese group may be different from our M112 originating from Romania. Our results indicate that the NWB CMS line may have originated from European wild radish. A previous study involving *Brassica napus* demonstrated the existence of a male-sterile line and its restorer line at the center of origin, whereas the maintainer lines always exist in populations far away from the center of origin [40]. This may help to explain why we identified three restorer lines following the extensive crossing with 16 radish inbred lines of European small radish (Table S1), whereas Nahm et al. failed to detect any restorer lines when they crossed the NWB CMS line with 58 breeding lines that were mainly East Asian long radish accessions [18]. Additionally, DCGMS mitotypes are predominantly in germplasm introduced from eastern European countries [41]. Most breeding lines bred in Korea were identified as maintainers of DCGMS and multiple *Rf* genes might exist in radish germplasm from European countries [42]. Both NWB CMS and DCGMS lines appear to have the same origin.

Regarding transcriptional regulation, the *orf463a* expression level was either upregulated or downregulated in the presence of *Rf* genes (Figure 6B). However, *orf463* transcription is reportedly unaffected by the presence of the dominant nuclear *Rf* gene in the DCGMS line [20]. This discrepancy may be because the analyzed flower buds were at different developmental stages in the two studies. Abnormal pollen abortion only occurs after the microspore stage and *orf463* expression may differ before and after the microspore stage [19,34]. Moreover, the existence of more than one *Rf* gene is supported by our results and those of studies on DCGMS. Thus, the *Rf* gene in the current study may be different from that in the DCGMS study [42]. For the crosses between the NWB CMS line and the Hongbaoshi, NWB CMS and Fure lines, male-sterile plants were not detected in the F_2_ populations, which is in contrast to the cross between NWB CMS and Rudi lines, which resulted in male-sterile plants in the F_2_ populations at a ratio of 1:15 (male-sterile:male-fertile; Table S1). These results indicate there are at least three *Rf* genes in the NWB CMS mitogenome, which is consistent with the findings of the Kim et al. (2010) study on DCGMS [42]. Finally, different *Rf* genes may act in distinct ways either at the transcriptional or post-transcriptional level (e.g., *Rf3* and *Rf4* in rice) [3]. The mechanism underlying the effects of *orf463a* on the CMS in the NWB CMS line and the interactions with nuclear genes are complex and need to be further characterized. Newly developed technologies, such as TALEN-mediated mitochondrial genome editing, may be useful for future investigations of CMS [43].

## 5. Conclusions

In this study, we interpreted the mitochondrial genomes of NWB CMS and Normal type, identified *orf463a* was the most likely causal factor for male sterility in NWB cytoplasm. Moreover, we found that NWB CMS and DCGMS are coexisting isomeric forms belonging to the same mitotype. *orf463a* was more highly expressed in flower buds than in vegetative organs and its expression was differentially regulated in the presence of restorer of fertility (*Rf*) genes. In addition, *orf463a* was confirmed to originate from *Raphanus raphanistrum*.

## Figures and Tables

**Table 1:** Sequence variations between the normal and NWB CMS mitochondrial genes encoding known proteins.

Gene	Position from Start Codon (on CDS)	Nucleotide Variation (Normal-NWB CMS)	Amino Acid Change (Normal-NWB CMS)
*coxI*	117	T-G	Synonymous
	207	T-C	Synonymous
*rps3*	551	G-A	G-E
*rps4*	1052	T-C	L-P
*rps12*	9	A-G	Synonymous
*rpl2*	204	G-A	Synonymous
	94	A-C	N-H
	93	A-T	K-N
	92	A-T	K-I
	91	A-T	K-I
	90	G-T	Synonymous
*matR*	210	T-C	Synonymous
	488	C-T	T-I
*nad7*	685	G-A	V-I

**Table 2:** Repeat sequences identified in the NWB CMS mitogenome.

Name	Length	Copy	Start–End	Junctions
Large repeat	9731	Copy 1	2–9732	1-2-Unique region
		Copy 2	196,094–205,824	10-12-11-13
R1	508	Copy 1	121,189–121,696	8-7-14
		Copy 2	215,328–215,835	Unique region-6
R2	307	Copy 1	51,926–52,232	3-Unique region
		Copy 2	80,333–80,639	15
R3	232	Copy 1	164–395	1
		Copy 2	112,845–11,3076	8
		Copy 3	196,256–196,487	10
R4	152	Copy 1	121,312–121,463	7
		Copy 2	162,014–162,165	Unique region
R5	147	Copy 1	83,785–83,931	15-16
		Copy 2	95,246–95,392	16-17
R6	141	Copy 1	101,517–101,657	17-8
		Copy 2	165,607–165,747	Unique region-18
R7	98	Copy 1	50,042–50,139	3
		Copy 2	121,275–121,372	8-7
		Copy 3	215652–215749	Unique region
R8	87	Copy 1	60,857–60,943	4
		Copy 2	117,566–117,652	8
R9	81	Copy 1	86–166	1
		Copy 2	125–205	1
		Copy 3	196,178–196,258	18-10
		Copy 4	196,217–196,297	18-10

**Table 3:** Details regarding 16 unique *orf* genes.

Features of the ORF	*orf* ID
In the unique region	***orf84h****, orf84g, orf82g, orf77f, **orf306a**, **orf72f***, ***orf71i****, **orf273a**, orf124a, **orf91a**, **orf463a**, orf86b*
In the non-unique region	*orf148a, orf79e, orf85f, orf85e*
Chimeric structure	*orf306a, orf72f, orf71i, orf273a, orf91a*
Transmembrane domain	*orf84h, orf306a, orf273a, orf463a*

**Table 4:** Distribution of *orf463a* in different radish species.

Species	Total Accessions	*orf463a*
*R. sativus var. hortensis* Becker (East Asian big long radish)	52	1
*R. sativus var. niger* Kerner (Black Spanish radish)	6	6
*R. sativus var. caudatus* Hooker and Anderson (tail-podded radish or rat-tail radish)	2	0
*R. sativus var. oleiferus* Metzg (oil radish)	1	0
*R. sativus var. sativus L.* (European small radish)	22	0
*R. sativus var. raphanistroides* Makino (East Asian wild radish)	3	0
*R. raphanistrum*	2	1
*R. maritimus*	4	1
*R. landra*	1	0

## Supplementary Materials

The following are available online at https://www.mdpi.com/2073-4425/11/1/74/s1. Table S1: Inheritance patterns of fertility restoration for male sterility caused by the NWB cytoplasm. Table S2: Primers used in this study. Table S3: Details regarding the assembled sequences. Table S4: Details regarding the non-coding RNA. Table S5: Types and number of tRNAs. Table S6: Homology matrix of 10 sequences using approximately 40 kb syntenic block. Table S7: Position of known or putative protein-coding genes in the NWB CMS mitochondrial genome. Table S8: Information regarding the indels among three mitogenomes. Figure S1: Alignment of ATP9 and ATP9-D2 amino acid sequences. Figure S2: Amplification of *orf463a* in 93 radish accessions. Sample numbers in the gel image correspond to the accession numbers in an article by Wang et al. (2015). Figure S3: Alignment of *orf463a* sequences from nine radish accessions.

## Abbreviations

CMS	Cytoplasmic male sterility
Rf	Restorer of fertility
ORF	Open reading frame
DCGMS	Dongbu cytoplasmic and genic male sterility
NGS	Next-generation sequencing
SNP	Single nucleotide polymorphism
InDel	Insertion–deletion
tRNA	Transfer RNA
rRNA	Ribosome RNA

## References

[1] Curtis I.S. (2011). Genetic engineering of radish: Current achievements and future goals. Plant Cell Rep..

[2] Hanson M.R., Bentolila S. (2004). Interactions of mitochondrial and nuclear genes that affect male gametophyte development. Plant Cell.

[3] Luo D., Xu H., Liu Z., Guo J., Li H., Chen L., Fang C., Zhang Q., Bai M., Yao N. (2013). A detrimental mitochondrial-nuclear interaction causes cytoplasmic male sterility in rice. Nat. Genet..

[4] Davila J., Arrieta-Montiel M., Wamboldt Y., Cao J., Hagmann J., Shedge V., Xu Y.Z., Weigel D., Mackenzie S.A. (2011). Double-strand break repair processes drive evolution of the mitochondrial genome in Arabidopsis. BMC Biol..

[5] Okazaki M., Kazama T., Murata H., Motomura K., Toriyama K. (2013). Whole mitochondrial genome sequencing and transcriptional analysis to uncover an RT102-type cytoplasmic male sterility-associated candidate gene derived from *Oryza rufipogon*. Plant Cell Physiol..

[6] Sarria R., Lyznik A., Vallejos C.E., Mackenzie S.A. (1998). A cytoplasmic male sterility-associated mitochondrial peptide in common bean is post-translationally regulated. Plant Cell.

[7] Wise R.P., Bronson C.R., Schnable P.S., Horner H.T. (1999). The genetics, pathology, and molecular biology of T-cytoplasm male sterility in maize. Adv. Agron..

[8] Koizuka N., Imai R., Fujimoto H., Hayakawa T., Kimura Y., Kohno-Murase J., Sakai T., Kawasaki S., Imamura J. (2003). Genetic characterization of a pentatricopeptide repeat protein gene, *orf687*, that restores fertility in the cytoplasmic male-sterile Kosena radish. Plant J..

[9] Kazama T., Nakamura T., Watanabe M., Sugita M., Toriyama K. (2008). Suppression mechanism of mitochondrial ORF79 accumulation by Rf1 protein in BT-type cytoplasmic male sterile rice. Plant J..

[10] Yamamoto M.P., Shinada H., Onodera Y., Komaki C., Mikami T., Kubo T. (2008). A male sterility-associated mitochondrial protein in wildbeets causes pollen disruption in transgenic plants. Plant J..

[11] Hanson M.R. (1991). Plant mitochondrial mutations and male sterility. Annu. Rev. Genet..

[12] Chen L., Liu Y. (2014). Male Sterility and Fertility Restoration in Crops. Annu. Rev. Plant Biol..

[13] Ogura H. (1968). Studies on the new male-sterility in Japanese radish, with special reference to the utilization of this sterility towards the practical raising of hybrid seeds. Mem. Fac. Agric. Kagoshima Univ..

[14] Krishnasamy S., Makaroff C.A. (1994). Organ-specific reduction in the abundance of a mitochondrial protein accompanies fertility restoration in cytoplasmic male-sterile radish. Plant Mol. Biol..

[15] Bonhomme S., Budar F., Férault M., Pelletier G. (1991). A 2.5 kb NcoI fragment of Ogura radish mitochondrial DNA is correlated with cytoplasmic male-sterility in Brassica cybrids. Curr. Genet..

[16] Bonhomme S., Budar F., Lancelin D., Small I., Defrance M.C., Pelletier G. (1992). Sequence and transcript analysis of the Nco2.5 Ogura-specific fragment correlated with cytoplasmic male sterility in Brassica cybrids. Mol. Gen. Genet..

[17] Yamagishi H., Terachi T., Nishi T., Kitashiba H. (2017). Cytoplasmic male sterility and mitochondrial genome variations in radish. The Radish Genome.

[18] Nahm S.H., Lee H.J., Lee S.W., Joo G.Y., Harn C.H., Yang S.G., Min B.W. (2005). Development of a molecular marker specific to a novel CMS line in radish (*Raphanus sativus* L.). Theor. Appl. Genet..

[19] Lee Y., Park S., Lim C., Kim H., Lim H., Ahn Y., Sung S., Yoon M., Kim S. (2008). Discovery of a novel cytoplasmic male-sterility and its restorer lines in radish (*Raphanus sativus* L.). Theor. Appl. Genet..

[20] Park J.Y., Lee Y.P., Lee J., Choi B.S., Kim S., Yang T.J. (2013). Complete mitochondrial genome sequence and identification of a candidate gene responsible for cytoplasmic male sterility in radish (*Raphanus sativus* L.) containing DCGMS cytoplasm. Theor. Appl. Genet..

[21] Yamagishi H., Tanaka Y., Shiiba S., Hashimoto A., Fukunaga A. (2019). Mitochondrial orf463 causing male sterility in radish is possessed by cultivars belonging to the ‘Niger’ group. Euphytica.

[22] Wang Q.B., Zhang L., Zheng P. (2015). Genetic diversity and evolutionary relationship analyses within and among *Raphanus* species using EST-SSR markers. Mol. Breed..

[23] Chen J., Guan R., Chang S., Du T., Zhang H., Xing H. (2011). Substoichiometrically different mitotypes coexist in mitochondrial genomes of *Brassica napus* L.. PLoS ONE.

[24] Borgstrom E., Lundin S., Lundeberg J. (2011). Large scale library generation for high throughput sequencing. PLoS ONE.

[25] Dmitry A., Anton K., Jeffrey S.M., Pavel A.P. (2016). HYBRIDSPADES: An algorithm for hybrid assembly of short and long reads. Bioinformatics.

[26] Haas B.J., Salzberg S.L., Zhu W., Pertea M., Allen J.E., Orvis J., White O., Buell C.R., Wortman J.R. (2008). Automated eukaryotic gene structure annotation using EvidenceModeler and the Program to Assemble Spliced Alignments. Genome Biol..

[27] Lowe T.M., Eddy S.R. (1997). tRNAscan-SE: A program for improved detection of transfer RNA genes in genomic sequence. Nucl. Acids Res..

[28] Lagesen K., Hallin P., Rødland E.A., Staerfeldt H.H., Rognes T., Ussery D.W. (2007). RNAmmer: Consistent and rapid annotation of ribosomal RNA genes. Nucl. Acids Res..

[29] Lohse M., Drechsel O., Bock R. (2007). OrganellarGenomeDRAW (OGDRAW): A tool for the easy generation of high-quality custom graphical maps of plastid and mitochondrial genomes. Curr. Genet..

[30] Kumar S., Stecher G., Li M., Knyaz C., Tamura K. (2018). MEGA X: Molecular Evolutionary Genetics Analysis across computing platforms. Mol. Biol. Evol..

[31] Saitou N., Nei M. (1987). The neighbor-joining method: A new method for reconstructing phylogenetic trees. Mol. Biol. Evol..

[32] Tamura K., Peterson D., Peterson N., Stecher G., Nei M., Kumar S. (2011). MEGA5: Molecular evolutionary genetics analysis using maximum likelihood, evolutionary distance, and maximum parsimony methods. Mol. Biol. Evol..

[33] Krogh A., Larsson B., von Heijne G., Sonnhammer E.L. (2001). Predicting transmembrane protein topology with a hidden Markov model: Application to complete genomes. J. Mol. Biol..

[34] Zhang L., Wang Q.B., Zheng P.J. (2014). Identification and Classification of different male sterile cytoplasms in radish. Acta Agric. Boreali Sin..

[35] Xu Y.Y., Zhu X.W., Gong Y.Q., Xu L., Wang Y., Liu L.W. (2012). Evaluation of reference genes for gene expression studies in radish (*Raphanus sativus* L.) using quantitative real-time PCR. Biochem. Biophys. Res. Commun..

[36] Tanaka Y., Tsuda M., Yasumoto K., Yamagishi H., Terachi T. (2012). A complete mitochondrial genome sequence of Ogura-type male-sterile cytoplasm and its comparative analysis with that of normal cytoplasm in radish (*Raphanus sativus* L.). BMC Genom..

[37] Gabay-Laughnan S., Newton K.J., Bock R., Knoop V. (2012). Plant mitochondrial mutations. Genomics of Chloroplasts and Mitochondria.

[38] Chang S., Chen J., Wang Y., Gu B., He J., Chu P., Guan R. (2013). The mitochondrial genome of *Raphanus sativus* and gene evolution of Cruciferous mitochondrial types. J. Genet. Genom..

[39] Shen D., Sun H., Huang M., Zheng Y., Qiu Y., Li X., Fei Z. (2013). Comprehensive analysis of expressed sequence tags from cultivated and wild radish (*Raphanus* spp.). BMC Genom..

[40] Fu T.D. (1989). Relationship between the origin and evolution of rapeseeds and the development of cytoplasmic male sterile “three lines”. Chin. J. Oil Crop Sci..

[41] Kim S., Lee Y., Lim H., Ahn Y., Sung S. (2009). Identification of highly variable chloroplast sequences and development of cpDNA-based molecular markers that distinguish four cytoplasm types in radish (*Raphanus sativus* L.). Theor. Appl. Genet..

[42] Kim K., Lee Y.P., Lim H., Han T., Sung S.K., Kim S. (2010). Identification of *Rfd1*, a novel restorer-of-fertility locus for cytoplasmic male-sterility caused by DCGMS cytoplasm and development of simple PCR markers linked to the *Rfd1* locus in radish (*Raphanus sativus* L.). Euphytica.

[43] Kazama T., Okuno M., Watari Y., Yanase S., Koizuka C., Tsuruta Y., Sugaya H., Toyoda A., Itoh T., Tsutsumi N. (2019). Curing cytoplasmic male sterility via Talen-mediated mitochondrial genome editing. Nat. Plants.

