# Supplementary material for: Mitochondrial Genome Sequencing Reveals orf463a May Induce Male Sterility in NWB Cytoplasm of Radish
Source: Genes (Basel). 2020 Jan 9;11(1):74. doi: 10.3390/genes11010074 (PMC7017215; doi:10.3390/genes11010074)
Supplement: Supplementary file 1 [file genes-11-00074-s001.zip › modified supplementary files/Supplementary tables.docx]

**Supplementary materials**

**Journal: Genes**

**Title: Mitochondrial genome sequencing reveals orf463a may induce male sterility in NWB cytoplasm of radish**

**Authors:** Yanping Wang^1,&^, Qingbiao Wang^1,&^, Wei Hao^1^, Jianxia Li^1^, Meixia Qi^1^, Li Zhang^1,*^

**Institution:** 1 National Engineering Research Center for Vegetables, Beijing Academy of Agriculture and Forestry Sciences, Key Laboratory of Biology and Genetic Improvement of Horticultural Crops (North China), Beijing Key Laboratory of Vegetable Germplasm Improvement, Beijing 100097, China

^*^Correspondence:

Email: zhangli@nercv.org

Tel.: +86 10 51503163

Address: 50 Zhanghua Road, Haidian District, Beijing, China

&:These authors contribute equally to this work.

Table S1 Inheritance patterns of fertility restoration of male-sterility caused by the NWB cytoplasm

| Populations | Expected (MF:MS) | Observed (MF:MS) | χ^2^ | p |
| --- | --- | --- | --- | --- |
| YB-A ×Hongbaoshi |  |  |  |  |
| F1 | 1:0 | 15:0 | 0 | 1 |
| F2 (SA18-14) | 3:1 | 96:0 | 32 | <0.001 |
| YB-A ×Fure |  |  |  |  |
| F1 | 1:0 | 15:0 | 0 | 1 |
| F2 (SA18-15) | 3:1 | 98:0 | 32 | <0.001 |
| YB-A ×Rudi |  |  |  |  |
| F1 | 1:0 | 15:0 | 0 | 1 |
| F2 (SA18-16) | 15:1 | 92:6 | 0.003 | 0.958 |
| YB-A × YB-B | 0:1 | 0:15 | 0 | 1 |
| YB-A ×Duanye 13 | 0:1 | 0:15 | 0 | 1 |
| YB-A ×Qingbaoxia | 0:1 | 0:15 | 0 | 1 |
| YB-A ×YRTengu | 0:1 | 0:15 | 0 | 1 |
| YB-A ×Xixing No.5 | 0:1 | 0:15 | 0 | 1 |
| YB-A ×Weiqing | 0:1 | 0:15 | 0 | 1 |
| YB-A × JZ wujinhong | 0:1 | 0:15 | 0 | 1 |
| YB-A ×Manshenhong | 0:1 | 0:15 | 0 | 1 |
| YB-A×BanyeChunlihong | 0:1 | 0:15 | 0 | 1 |
| YB-A×GangShui | 0:1 | 0:15 | 0 | 1 |

MF: Male fertile; MS: Male sterile

Table S2Primers used in this study

| Gene | Forward primer (5ˊ- 3ˊ) | Reverse primer (5ˊ- 3ˊ) |
| --- | --- | --- |
| Primers used for ampification of *orfs* and COXI | | |
| *orf84h* | ATGTGTCGTGTTCCTTTGGCC | CTAAATCCTGAAGAAAGCTAGAAAG |
| *orf306a* | ATGTATCTACTTATCGTATTTTTAC | TTAGTTTTTGGGGGGTTGGTG |
| *orf72f* | ATGACTTTGAACTTCGAGTTCTCGA | CTATTTCGTTGTACAACGACGAGAG |
| *orf71i* | ATGGGATCAAAAACAACCTATCACC | TTAGGGAGACACGCGCGCAT |
| *orf273a* | ATGCGAAGAAATCTAAATCTCCTT | TCAGAATACGAATAAGATCAAAAAG |
| *orf91a* | ATGCGCGTTAGCGCAACGGCTTTCG | TCAATGCATTCGTACCAGGAGGTAA |
| Primers used for ampification of *orfs* and COXI | | |
| *orf463* | TGCTTTGGCAGGAGCTGCAAGCTATTT | AGGACACTTTTTAGCATGCCGGAGCCT |
| *COXI* | ATGAAAAATCTGGTTCGATGGCTGT | TTACTTCACATAGCTTTTCGTCTCC |
| Real time PCR primers | | |
| *RPII* | CTCTCTGGGGCTTTAGCCATCTTCT | GGTCATTGTCCTGACGGTTCTGAAT |
| *orf463a* | CTCTCTGGGGCTTTAGCCATCTTCT | GGTCATTGTCCTGACGGTTCTGAAT |
| Primers used for verify the master and subgenomic circles in NWB CMS | | |
| 5+4 (MC1) | TCTCCGAAACAAGAGATCAAAGTG | TTCCGTTTCCACAACGTGTTGA |
| 2+3 (MC1) | TTGGTTGTTGACCAACAAAAGCAT | GTGCCCATCACTCCAGCAATG |
| 5+3 (MC2) | TCTCCGAAACAAGAGATCAAAGTG | GTGCCCATCACTCCAGCAATG |
| 4+2 (MC2) | TTGGTTGTTGACCAACAAAAGCAT | TTCCGTTTCCACAACGTGTTGA |
| 1+5 (MC3) | GATCTCTTCACTGGATGTGTCGTGT | CCACAAGAGTATACTCATTTCCGCA |
| 2+3 (MC3) | GGGAAGAAACTCTCTTTGACTTTTG | CAGCGGAGCGTAACCACTTTCT |
| 3+4 (SC1) | GTGCCCATCACTCCAGCAATG | TTCCGTTTCCACAACGTGTTGA |
| 2+5 (SC2) | TCTCCGAAACAAGAGATCAAAGTG | TTGGTTGTTGACCAACAAAAGCAT |
| 3+5 (SC3) | CAGCGGAGCGTAACCACTTTCT | CCACAAGAGTATACTCATTTCCGCA |
| 1+2 (SC4) | GGGAAGAAACTCTCTTTGACTTTTG | GATCTCTTCACTGGATGTGTCGTGT |

Table S3 Statistic of assembling

| Sample ID | Sequence Number(#) | Total Length(bp) | | GC Content(%) | N rate (%) |
| --- | --- | --- | --- | --- | --- |
| Normal | 1 | | 239725 | 45.26% | 0% |
| NWBCMS | 1 | | 239195 | 45.13% | 0% |

N rate(%)：The content of unknown base N.

Table S4 Statistics of non-coding RNA (ncRNA)

| Sample ID | ncRNA type | ncRNA number (#) | ncRNA average length (bp) | ncRNA total length (bp) | ncRNA length / Genome (%) |
| --- | --- | --- | --- | --- | --- |
| Normal | tRNA | 24 | 77 | 1,868 | 0.7792 |
|  | 5S | 1 | 119 | 119 | 0.0496 |
|  | 18S | 1 | 1848 | 1848 | 0.7709 |
|  | 26S | 1 | 3176 | 3176 | 1.3249 |
| NWBCMS | tRNA | 25 | 77 | 1937 | 0.8098 |
|  | 5S | 1 | 119 | 119 | 0.0498 |
|  | 18S | 1 | 1848 | 1848 | 0.7726 |
|  | 26S | 1 | 3176 | 3176 | 1.3279 |

Table S5 Types and number of tRNA

| Normal | tRNA(type) | ASN | ASP | CYS | GLN | GLU | GLY | HIS | IIE | LEU | LYS | MET | PRO | SER | THR | TYR | UNDET | TRP | Total |
| --- | --- | --- | --- | --- | --- | --- | --- | --- | --- | --- | --- | --- | --- | --- | --- | --- | --- | --- | --- |
|  | Number | 1 | 1 | 1 | 1 | 1 | 1 | 1 | 1 | 1 | 1 | 3 | 1 | 3 | 1 | 4 | 1 | 1 | 24 |
| NWBCMS | tRNA(type) | ASN | ASP | CYS | GLN | GLU | GLY | HIS | IIE | LEU | LYS | MET | PRO | SER | THR | TYR | UNDET | TRP | Total |
|  | Number | 1 | 1 | 1 | 1 | 1 | 1 | 1 | 1 | 1 | 1 | 4 | 1 | 3 | 1 | 4 | 1 | 1 | 25 |

Table S6 Homology matrix of 10 sequences using approximately 40-kb syntenic block

| AB694743 Uchiki-gensuke | 100% |  |  |  |  |  |  |  |  |  |
| --- | --- | --- | --- | --- | --- | --- | --- | --- | --- | --- |
| AB694744 MS-gensuke | 90.5% | 100% |  |  |  |  |  |  |  |  |
| AP012990 Black radish | 90.4% | 100.0% | 100% |  |  |  |  |  |  |  |
| AP012991 OS40 | 90.4% | 99.9% | 99.9% | 100% |  |  |  |  |  |  |
| AP013077 Aonaga | 99.9% | 87.5% | 87.4% | 87.4% | 100% |  |  |  |  |  |
| JQ083668 Chuanxinhong | 99.9% | 87.5% | 87.4% | 87.4% | 100.0% | 100% |  |  |  |  |
| KC193578 DCGMS | 90.5% | 100.0% | 100.0% | 99.9% | 87.5% | 87.5% | 100% |  |  |  |
| KJ716484 WK10039 | 100.0% | 90.5% | 90.4% | 90.4% | 99.9% | 99.9% | 90.5% | 100% |  |  |
| Normal | 99.9% | 87.5% | 87.4% | 87.4% | 100.0% | 100.0% | 87.5% | 99.9% | 100% |  |
| NWB CMS | 90.5% | 100.0% | 100.0% | 99.9% | 87.5% | 87.5% | 100.0% | 90.5% | 87.5% | 100% |
