# Supplementary figures and images for: Mitochondrial Genome Sequencing Reveals orf463a May Induce Male Sterility in NWB Cytoplasm of Radish
Source: Genes (Basel). 2020 Jan 9;11(1):74. doi: 10.3390/genes11010074 (PMC7017215; doi:10.3390/genes11010074)

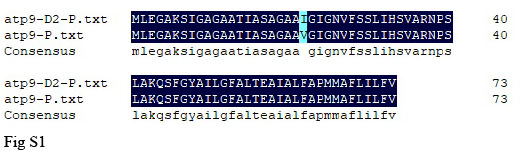

Supplement: Supplementary file 1 [file genes-11-00074-s001.zip › modified supplementary files/Fig S1.jpg]

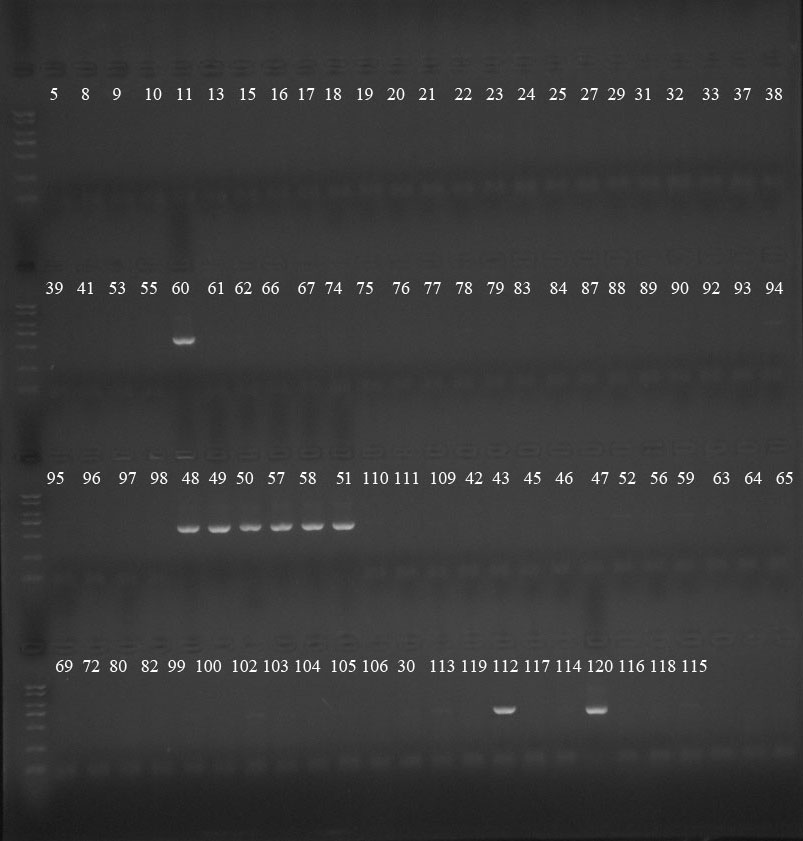

Supplement: Supplementary file 1 [file genes-11-00074-s001.zip › modified supplementary files/Fig S2.jpg]

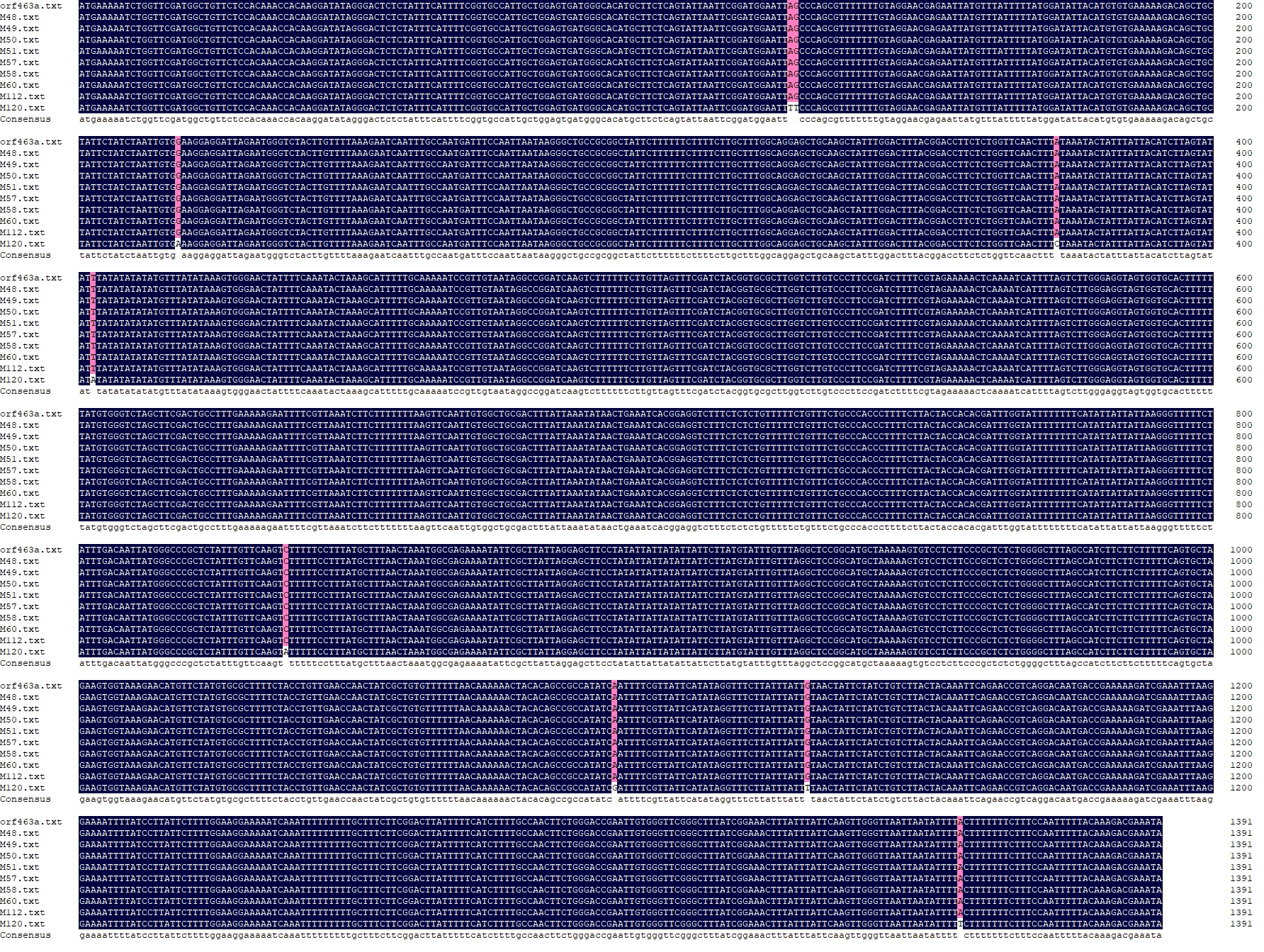

Supplement: Supplementary file 1 [file genes-11-00074-s001.zip › modified supplementary files/Figure S3.jpg]
